# Draft genome sequence of *Leuconostoc falkenbergense* isolated from naturally fermented buffalo milk curd

**DOI:** 10.1128/mra.00148-24

**Published:** 2024-04-11

**Authors:** Mst. Umme Habiba, M. Nazmul Hoque, Shabbir Ahmed, Tofazzal Islam, Gautam Kumar Deb, Md. Morshedur Rahman

**Affiliations:** 1 Department of Dairy and Poultry Science, Bangabandhu Sheikh Mujibur Rahman Agricultural University (BSMRAU), Gazipur, Bangladesh; 2 Molecular Biology and Bioinformatics Laboratory, Department of Gynecology, Obstetrics and Reproductive Health, Bangabandhu Sheikh Mujibur Rahman Agricultural University (BSMRAU), Gazipur, Bangladesh; 3 Institute of Biotechnology and Genetic Engineering, Bangabandhu Sheikh Mujibur Rahman Agricultural University (BSMRAU), Gazipur, Bangladesh; 4 Biotechnology Division, Bangladesh Livestock Research Institute, Savar, Dhaka, Bangladesh; 5 Institute of Food Safety and Processing, Bangabandhu Sheikh Mujibur Rahman Agricultural University (BSMRAU), Gazipur, Bangladesh; Department of Biological Sciences, Wellesley College, Wellesley, Massachusetts, USA

**Keywords:** whole genome, *Leuconostoc falkenbergense*, buffalo milk curd

## Abstract

This study reports the draft genome of *Leuconostoc falkenbergense* strain BSMRAU-M1L5, isolated from artisanal buffalo milk curd in Bangladesh. The draft genome spans 1,776,471 bp, with 50× coverage and 96 contigs.

## ANNOUNCEMENT

*Leuconostoc*, a versatile genus of lactic acid bacteria, excels in diverse environments like plant matrices and fermented foods, playing a vital role in fermentation (1). We employed whole-genome sequencing (WGS) to unveil new insights into the genome of *Leuconostoc falkenbergense* strain BSMRAU-M1L5 (formerly *Leuconostoc pseudomesenteroides)* (2). *L. falkenbergense* BSMRAU-M1L5 was isolated from naturally fermented buffalo milk curd available in *Bhola* (22.19°N, 90.76°E) district of Bangladesh. After serial dilutions of locally manufactured buffalo milk curd samples, the culture-dependent isolation was conducted using modified milk plate count agar containing glucose as a carbon source, supplemented with vancomycin, and incubated aerobically at 22°C for 5 days (3). A purified single colony obtained from subsequent subculture was further grown in de Man, Rogosa, and Sharpe (MRS) broth for 48 h at 22°C. Afterward, genomic DNA was extracted using QIAamp DNA Mini Kit (QIAGEN, Hilden, Germany) (4). Purity and concentration of the extracted DNA were checked by NanoDrop 2000 UV-Vis Spectrophotometer (Thermo Fisher, USA). Libraries for WGS were prepared from 1 ng of DNA using the Nextera DNA Flex Library Prep Kit (Illumina, USA), and paired-end (2 × 250 bp protocol) sequencing was done with Illumina MiSeq sequencer (4, 5).

Raw reads (*n* = 9,552,456 bp) were trimmed for quality and adaptor sequence by Trimmomatic v.0.39 (6), and quality of reads was checked using FastQC v.0.11.9 (7). The contig-level genome assembly was carried out with SPAdes v.3.12 1 (8), and assembled genome was quality checked through QUAST v.5.0.2 (9). The draft genome annotation was performed by the National Center for Biotechnology Information Prokaryotic Genome Annotation Pipeline v.6.6 (10). We utilized ResFinder v.4.4.2 (11), PlasmidFinder (12), PHASTER (https://phaster.ca/), and RAST FIGfams v.70 (13) databases to predict antimicrobial resistance genes, plasmid, prophages, and metabolic functions, respectively, in the draft genome. BAGEL4 webserver (14) was used to predict bacteriocin gene clusters and secondary metabolite biosynthetic gene clusters. Default parameters were employed for all software unless explicitly specified.

The draft genome of BSMRAU-M1L5 consists of 96 contigs with a length of 1,776,471 bp. The draft genome has a G + C content of 39.2% and 50× coverage. The largest contig was 309,038 bp, with *N*_50_ and *L*_50_ values of 41,680 and 10, respectively. There are 1,763 protein-coding sequences, including 38 tRNAs, 3 rRNA operons, three noncoding RNAs, and 39 pseudogenes. The draft genome harbored 23 genes encoding for resistance to antibiotics and toxic compounds, including three genes conferring resistance to vancomycin (e.g., *van*B, *van*W, and *van*Y). The BSMRAU-M1L5 genome contained seven CRISPR arrays and nine prophages (with 95% identity and 60% coverage). No bacteriocin gene cluster and secondary metabolite biosynthetic gene were predicted in BSMRAU-M1L5 genome. These data will furnish valuable insights for researching the functions of this strain and will aid in the development of strains without vancomycin-resistance genes.

## Data Availability

The whole-genome shotgun project for the *Leuconostoc falkenbergense* BSMRAU-M1L5 has been submitted at GenBank under BioProject PRJNA982305. The raw sequence data were deposited in the SRA database under accession number SRR25321898. The version described in this paper is JAVUPV000000000.1.
